# Robotic Subtotal Cholecystectomy in a Geriatric Acute Care Surgery Patient with Super Obesity

**DOI:** 10.1155/2021/9992622

**Published:** 2021-06-21

**Authors:** Diane Bronikowski, Dominic Lombardo, Connie DeLa'O, Nova Szoka

**Affiliations:** West Virginia University, Department of Surgery, Morgantown, WV 26505, USA

## Abstract

*Introduction*. Unique challenges exist with conventional laparoscopic operations in patients with super obesity (BMI > 50). Limited literature is available regarding use of the robotic platform to treat patients with super obesity or acute care surgery patients. This case describes an interval robotic subtotal cholecystectomy in an elderly patient with super obesity and multiple comorbidities. *Case Description*. A 74-year-old male with a BMI of 59.9 developed acute cholecystitis. He was deemed excessively high risk for operative intervention due to concurrent comorbid conditions and underwent percutaneous cholecystostomy. After a few months, a cholangiogram demonstrated persistent cystic duct occlusion. The patient expressed interest in tube removal and elective interval cholecystectomy. After preoperative risk stratification and optimization, he underwent a robotic subtotal cholecystectomy with near infrared fluorescence cholangiography. The patient was discharged on postoperative day one and recovered without complications. *Discussion*. Obesity is a risk factor for acute cholecystitis, which is most commonly treated with conventional laparoscopy (CL). CL is technically restraining and difficult to perform in patients with super obesity. The body habitus of patients with super obesity can impair proper instrumentation and increase perioperative morbidity. In this case, robotic assisted cholecystectomy console improved surgeon ergonomics and provided support for proper instrumentation. Robotic, minimally invasive cholecystectomy approaches may reduce perioperative morbidity in patients with super obesity. Further studies are necessary to address the role of robotic surgery in acute care surgery patients with super obesity.

## 1. Introduction

The prevalence of adult obesity in the United States is 42.4%. Obesity is a risk factor for acute cholecystitis, which is most commonly treated with a laparoscopic cholecystectomy [1–4]. Challenges associated with conventional laparoscopy (CL) in patients with super obesity include adequate pneumoperitoneum, difficult trocar placement, surgeon fatigue, and increased risk of perioperative morbidity [5–7]. The rate of minimally invasive cholecystectomy is increasing in patients with obesity, despite the specific challenges associated with laparoscopic operations in this patient population. Limited literature is available regarding use of the robotic platform, an alternative approach, for patients with super obesity in the acute care setting. This case features a patient with super obesity who underwent a robotic assisted cholecystectomy (RAC) without the complications associated with CL.

## 2. Case Description

A 74-year-old male with super obesity (BMI of 59.9) presented to an outside facility with right upper quadrant pain that began two days prior. He had the following comorbidities: type II diabetes mellitus, coronary artery disease, congestive heart failure with ejection fraction of 40%, atrial fibrillation on long-term dual anticoagulation, sick sinus syndrome requiring implanted pacemaker, asthma, pulmonary hypertension, wheelchair dependence, and a 20 pack-year smoking history. Initial evaluation revealed leukocytosis and computed tomography scan findings of a distended gallbladder with inflammatory changes concerning for acute cholecystitis. Intravenous antibiotics were started, and the patient was transferred to our facility for further surgical evaluation and intervention.

**Figure 1:**
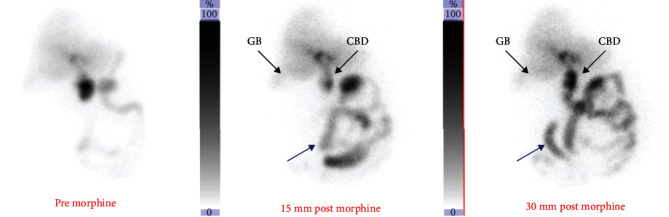
Hepatobiliary Iminodiacetic Acid scan images. Hepatobiliary scan images representative of acute cholecystitis. The right pictures show tracer filling the Common Bile Duct (CBD) and small bowel (blue arrow) and no tracer filling the gallbladder (GB) after morphine administration.

A Hepatobiliary Iminodiacetic Acid (HIDA) scan was performed which revealed acute acalculous cholecystitis (Figure 1). A percutaneous cholecystostomy tube (PCT) was placed by interventional radiology. The patient was discharged to home with planned interval cholangiograms and PCT checks. Subsequently, the patient had complications with his PCT including misplacement, occlusion, and pain. Three months after discharge, a cholangiogram demonstrated persistent cystic duct obstruction. The patient expressed interest in PCT removal and a cholecystectomy.

Preoperative evaluation by pulmonology revealed undiagnosed obstructive sleep apnea (OSA) and determined that the patient had an intermediate increased risk of perioperative complications. Evaluation by cardiology found that the patient had a moderate increased risk for cardiac complications with a laparoscopic cholecystectomy and a moderate to high risk with a laparotomy. After six weeks of appropriate therapy for OSA, the patient underwent a robotic assisted laparoscopic cholecystectomy.

**Figure 2:**
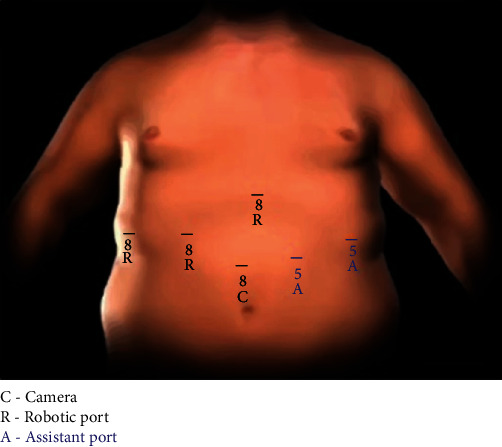
Diagram of robotic port placement. Diagram of robotic port placement with three robotic arm ports placed at the positions marked with a black “R” and the robotic camera port marked with a black “C.” Two laparoscopic assistant ports were placed at the positions marked with a blue “A.”

**Figure 3:**
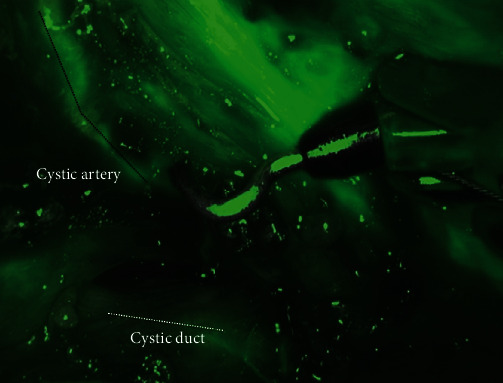
Indocyanine green dye to confirm correct identification of cystic artery. Image showing use of near infrared camera and indocyanine green dye to confirm correct identification of cystic artery marked with black dotted line and cystic duct marked with white dotted line.

Four robotic ports and two laparoscopic ports were placed (Figure 2). Dissection began with limited workspace due to patient body habitus and was further restricted by significant scarring surrounding the gallbladder. This workspace challenge was ameliorated after suspending the falciform ligament with a transfascial suture. Dissection exposed the cystic duct and cystic artery. Near infrared fluorescence cholangiography (NIRF-C) with injection of indocyanine green dye was performed to confirm the location of the cystic artery (Figure 3). The critical view of safety was obtained. The cystic artery and duct were clipped and divided, and the anterior wall of the gallbladder was removed along with the remaining gallstones. Due to significant inflammation, the posterior wall of the gallbladder was unable to be removed safely from the liver bed. The posterior gallbladder wall was cauterized to reduce the chance of bile leak, and a drain was placed in the gallbladder fossa. The patient did well postoperatively, was discharged home postoperative day one, and recovered without complications. Pathology revealed severe acute on chronic cholecystitis with cholelithiasis.

## 3. Discussion

The prevalence of adult obesity continues to rise. Obesity increases the risk for developing acute cholecystitis, which is most commonly treated with a laparoscopic cholecystectomy [1–4]. This poses a challenge for patients with super obesity (BMI > 50) because increased BMI is associated with adverse outcomes in laparoscopic procedures [5–7]. The alternative to conventional laparoscopy (CL), laparotomy, increases patient morbidity and mortality, independent of BMI [1, 8]. Further, one cohort of 20,979 demonstrated that laparoscopic converted to open (LCO) cholecystectomy occurred more in patients with super obesity, and CL was attempted less overall in lieu of laparotomy. With the rising rate of obesity, it is necessary to evaluate alternative options to CL and laparotomy. The challenges associated with CL in patients with super obesity and the benefits of robotic assisted cholecystectomy (RAC) are discussed below.

Specific challenges may arise during CL. Increased insufflation pressures may be required to combat the weight of the patient's abdominal wall. These pressures can decrease venous return and cardiac output and impede ventilation, necessitating higher inspiratory pressures and increasing the risk of barotrauma [9]. The body habitus of patients with super obesity can impair proper trocar placement which can restrict instruments and make dissection harder due to the fulcrum effect [10]. Additionally, increased muscle activity is required to complete the same tasks in patients with obesity compared to patients without resulting in more surgeon fatigue [11].

RAC eliminates the fulcrum effect and provides superior intraoperative dexterity. The robotic console improves surgeon ergonomics and provides 3D vision with true-depth perception. RAC reduces the need for increased insufflation pressures because the fixed robotic arms stabilize and support the abdominal wall in an elevated position [12]. RAC allows for better control, less surgeon fatigue, and more precise dissection [3, 10, 13]. In this case, the robotic approach facilitated case completion with a single surgeon and assistant using 4 robotic ports and 2 assistant ports whereas CL would have required two bedside assistants.

Robotic assisted laparoscopy has been demonstrated to have significantly lower overall complication rates, less blood loss, and shorter postoperative hospital stays when compared to CL in patients with obesity [7]. Robotic assistance can also reduce the rates of LCO and laparotomies in patients with obesity [12]. This case supports these findings.

An often-controversial topic of RAC is the expense with no significant difference in patient outcomes when compared to CL [13]. However, there is evidence of cost neutralization with utilization of robotics in patients with high BMIs solely due to the decreased rate of laparotomies and LCO [12].

In this case of a geriatric patient with super obesity and multiple comorbidities, RAC was utilized because of its 3D camera, normal insufflation pressures, and superior maneuverability. RAC reduced surgeon fatigue while minimizing patient intraoperative risks. Robotic, minimally invasive cholecystectomy approaches may reduce perioperative morbidity in patients with super obesity. Further studies are necessary to address the role of robotic surgery in acute care surgery patients with super obesity.

## 4. Conclusion

This case demonstrates effective use of robotic assisted cholecystectomy in an acute care surgery patient with super obesity while avoiding laparotomy and perioperative complications. This case can serve as a model for further studies exploring the safety, efficacy, and value of robotic assistance for patients with super obesity in the acute care setting.

## 5. Lessons Learned

Conventional laparoscopy is technically restraining and difficult to perform in patients with super obesity. Robotic assistance is an alternative with advantages that apply to both the surgeon and patient.

## Data Availability

No data were used to support this study.
